# Learning about protein solubility from bacterial inclusion bodies

**DOI:** 10.1186/1475-2859-8-4

**Published:** 2009-01-08

**Authors:** Mónica Martínez-Alonso, Nuria González-Montalbán, Elena García-Fruitós, Antonio Villaverde

**Affiliations:** 1 Institute for Biotechnology and Biomedicine and Department of Genetics and Microbiology, Autonomous University of Barcelona, Barcelona, Spain; 2 CIBER de Bioingeniería, Biomateriales y Nanomedicina (CIBER-BBN), Spain

## Abstract

The progressive solving of the conformation of aggregated proteins and the conceptual understanding of the biology of inclusion bodies in recombinant bacteria is providing exciting insights on protein folding and quality. Interestingly, newest data also show an unexpected functional and structural complexity of soluble recombinant protein species and picture the whole bacterial cell factory scenario as more intricate than formerly believed.

## Commentary

The conformational quality of soluble recombinant proteins is an emerging matter of concern, especially when the obtained products are to be used for functional or interactomic analyses [1]. In the context of recombinant protein production, the general believing that soluble protein species are properly folded and fully functional in contrast to the misfolded and inactive protein versions trapped in insoluble inclusion bodies [2], cannot be longer supported by current research data. The dropping of independent references to inclusion bodies as entities formed by functional protein species with native secondary structure is progressively increasing, and the structural and functional diversity of the model proteins used in these studies [3-13] does leave little room to speculate about this fact as being an artefact or a peculiarity of a limited number of protein species. Recent reviews in this area have presented properly folded proteins as natural components of inclusion bodies [10,14], indirectly compromising the paradigm of recombinant protein solubility as equivalent to protein conformational quality [15].

Indeed, the occurrence of functional proteins as important components of bacterial aggregates prompts to reconsider the conformational quality of protein species occurring in the soluble cell fraction of inclusion body-forming cells, that might be lower than expected. Several indirect observations are also in this line; (i) the functional quality of recombinant proteins in *E. coli *is affected in parallel by physical parameters such as temperature (high temperature impairs protein activity in both soluble and insoluble cell fractions) [16] and physiological conditions such as the availability of chaperones (a molar excess of DnaKJ inactivates both soluble and insoluble recombinant proteins) [17]; (ii) *in vivo* disintegration of inclusion bodies is strongly dependent on proteolytic degradation [18-21] for which DnaK is required [20], indicating a tight surveillance of the quality control system over aggregated protein species; (iii) inclusion body-forming proteins can complete their folding process once embedded in these aggregates [22]; (iv) the soluble versions of recombinant proteins can occur as soluble aggregates [23,24]; (v) the functional quality (measured for a model enzyme as its specific activity and fluorescent proteins by specific emission) of soluble protein versions can be lower than that of the inclusion body counterparts [3], and be eventually improved by reducing the growth temperature of recombinant cells from 37 to 16°C [16]. This indicates that at 37°C, an important fraction of soluble protein species are inactive, suggesting that they have not reached their native conformation. This has been very recently explored by sub-fractioning the soluble population of an inclusion body-forming recombinant GFP and their subsequent functional analysis. Indeed, there is a large functional diversity within the soluble protein population (accompanied by an extremely high abundance of soluble aggregates, either globular or fibrilar) [24], that prompts to observe the specific fluorescence of the soluble protein version as an average rather than a canonical value defined by a single type of molecular species.

In this scenario, recombinant proteins in producing cells can be seen as adopting "a continuum of forms" [23] expanding from soluble to insoluble cell fractions, and inclusion bodies as insoluble "clusters" of protein species [19]. Therefore, soluble versions of a given protein would not necessarily show better conformational quality than the aggregated counterparts, although the average biological activity (specific activity for enzymes or specific fluorescence for fluorescent proteins) is in general higher in the soluble cell fraction [3,24]. Interestingly, the specific enzymatic activities (or fluorescence emission) of soluble and insoluble protein versions tend to adopt similar values under specific conditions such as in DnaK knockout mutants [25,26]. Therefore, the soluble and insoluble "virtual" cell fractions in bacteria [14] are now regarded as more virtual than ever, as the main feature distinguishing soluble and inclusion body protein species might be the dispersed-clustered status rather than the biological activity.

From a practical point of view, these emerging concepts about protein aggregation in recombinant bacteria have remarkable implications. First, inclusion bodies formed by enzymes can be straightforward used as catalysers in industry-relevant enzymatic reactions skipping any previous *in vitro* refolding protocols [5-7]. Second, the quality of inclusion body proteins can be dramatically enhanced by producing them at suboptimal temperatures. This should not only permit the production of inclusion bodies with improved catalyzing properties but it also might favour the controlled *in vitro* release of functional proteins from these aggregates. In this regard, the recovery of functional proteins from inclusion bodies has been a largely used strategy when a desired protein species showed a high aggregation tendency. Such an approach implies separation of inclusion bodies, efficient protein unfolding under extreme denaturation conditions and further refolding through complex (and often unsuccessful) step strategies to be optimized for any particular protein species [27]. However, in the last years, an increasing piece of evidence points out that inclusion bodies with high content of native-like structure could be easily solubilised in non-denaturing conditions avoiding strong denaturation and refolding steps. A set of non related proteins, namely GFP [28], archaeon proteins, cytokines, immunoglobulin-folded proteins [29] and β-2-microglobulin [30], have been successfully extracted from inclusion bodies without the need of denaturing conditions, basically using as solubilising agents L-arginine and GdnHCl at non-denaturing concentrations [28,29]. Also in this line, Menart and co-workers observed that functional proteins could be easily extracted from inclusion bodies using non denaturing mild detergents and polar solvents, provided that the cells would have been cultured under suboptimal temperatures [12]. Such inclusion bodies, being a straightforward source of soluble proteins, were named "non-classical" because of their unexpected high content of functional, extractable species. Although sufficient data has been now accumulated to infer that in general, inclusion bodies are non-classical by nature (regarding the unlink between solubility and activity) [15], this interesting approach would potentially permit to skip complex refolding procedures by engineering the quality of inclusion body proteins during the production process. In very recent papers, Peternel and co-workers reported not only the successful extraction of functional polypeptides from inclusion bodies but also the fact that, in some cases, the biological activity of these inclusion body-solubilised proteins was comparable or even higher that than found in the soluble fraction. For instance, human granulocyte-stimulating factor (hG-CSF), GFP and lymphotoxin α (LT-α) extracted from inclusion bodies represented around the 98%, 40% and 25%, respectively, of the total biological activity and fluorescence emission in the recombinant protein producing-cells [31,32]. Again, the different structural and biological properties of the proteins for which this principle has been proved indicate that the extractability of functional proteins from inclusion bodies is not a particular issue, although its applicability at large scale needs to be further evaluated. On the other side, as an additional strategy, the specific activity of inclusion body proteins can be successfully enhanced by down-regulating the levels of recombinant gene expression [33,34].

Finally, since early recombinant DNA times, when the formation of inclusion bodies was noticed as a general undesirable event [35], enhancing protein solubility has been compulsory pursued through diverse approaches. The need for soluble proteins for many research, industrial and pharmaceutical applications has pushed microbiologists, biochemists and chemical engineers to modify cell, protein and process conditions (using protease-deficient cells, chaperone co-production, removing hydrophobic regions, fusion of solubility tags, minimizing the growth rate or using weak gene expression induction conditions among others), in an attempt to favour the occurrence of the target protein in the soluble cell fraction [36-41]. However, solubility is often observed as an academic parameter, namely the quotient (in %) between soluble and total protein and therefore with a questionable practical value. Interestingly, it is very rare to find in the literature measures of solubility simultaneous to determinations of protein yield or functional quality, when attempting a novel strategy to minimize inclusion body formation, such as for instance, the co-production of chaperones along with the recombinant protein species. In this regard, enhancing the levels of trigger factor and GroELS increases the solubility of a recombinant lysozyme that shows a specific activity lower than in absence of additional chaperones [42]. Other chaperone sets have been observed to promote solubility of target proteins [40,43,44] without a detailed analysis of protein quality and activity or by determining specific activity referring it to cell extracts or total (recombinant or not) protein [45,46]. Also, there are clear indications that the solubility enhancement under such conditions might eventually be associated to an increase of soluble aggregates [47]. Interestingly, lower protein yields obtained during chaperone co-production result in higher enzymatic activity in cell extracts and enhanced solubility, as observed by cyclodextrin glycosyltransferase [48] and mouse endostatin and human lysozyme respectively [42].

Furthermore, fine analyses of solubility in combination with other more useful parameters such as yield of soluble polypeptide or the biological activity reveal intriguing physiological events. For instance, co-production of the DnaKJ chaperone pair along with a target recombinant protein indeed favours solubility but at expenses of protein quality and yield [20]. In fact, enhancing the intracellular levels of DnaK, alone or within distinct chaperone sets (a common strategy to increase solubility) [40], dramatically diminishes protein stability through the stimulation of Lon- and ClpP-dependent proteolysis of inclusion body polypeptides [20]. In this regard, both yield and quality of a model recombinant GFP and other unrelated proteins are largely enhanced in DnaK^- ^mutants [20,25,26], in which the solubility percent value is, as expected, lower than in wilt type hosts. More intriguingly, plotting solubility percent data versus protein yield of functional quality renders extremely good but negative correlations, under different genetic backgrounds [20] or production conditions [49]. Preliminary data about non bacterial protein production systems from our group obtained by M. Martínez Alonso (not shown) indicate that such a negative correlation between yield (or quality) and solubility could be a general issue. Therefore, when designing a protein production process the most pertinent strategy should be chosen depending on what parameter (yield, quality or solubility) is the most relevant to the final use of the protein. Eventually, recombinant protein solubility could be merely dependent on the intracellular concentration of the recombinant protein itself, what would ultimately fit with the enhanced solubility observed at low growth rates, low temperatures and weak doses of gene expression inductor [36,41].

There are still exciting issues regarding bacterial inclusion bodies that deserve full scientific attention, such as the solving of the inner molecular organization that allows the occurrence of proper folded species within a general amyloid-like aggregate pattern [50,51]. Also, the sequence-dependent nature of protein aggregation [52-54] is still poorly known from a mechanistic point of view. From the biotechnological side, it is widely accepted that production of aggregation-prone protein triggers cell responses to conformational stress [55-58], irrespective of the host used as cell factory [59]. If such set of physiological responses cannot be efficiently controlled, enhancing protein solubility without renouncing to protein quality might be then a mirage. Surfing the complex network of cell activities that regulate protein aggregation (for instance, through rational metabolic engineering) could be a choice strategy to approach the production of soluble and high quality recombinant proteins. For such a more gentle use of cell factories, a deeper comprehension of the recombinant cell physiology and quality control system is urgently needed.

## Competing interests

The authors declare that they have no competing interests.

## Authors' contributions

MMA, NGM and EGF have equally contributed to this work.
